# Successful venous thromboprophylaxis in a patient with vaccine-induced immune thrombotic thrombocytopenia (VITT): a case report of the first reported case in Thailand

**DOI:** 10.1186/s12959-021-00317-3

**Published:** 2021-09-08

**Authors:** Archrob Khuhapinant, Tarinee Rungjirajittranon, Bundarika Suwanawiboon, Yingyong Chinthammitr, Theera Ruchutrakool

**Affiliations:** Division of Hematology, Department of Medicine, Faculty of Medicine Siriraj Hospital, Mahidol University, 2 Wanglang Road, Bangkok Noi, Bangkok, 10700 Thailand

**Keywords:** COVID-19 vaccine, Thrombocytopenia, Thrombosis, Vaccine-induced immune thrombotic thrombocytopenia

## Abstract

**Background:**

Vaccine-induced immune thrombotic thrombocytopenia (VITT) is a rare but fatal complication of the Coronavirus Disease 2019 vaccine. The many reports of VITT have mostly been in the Caucasian population. Here, we present the first reported case in an Asian population.

**Case presentation:**

A 26-year-old female had severe headache and severe thrombocytopenia 8 days after administration of the ChAdOx1 nCoV-19 vaccine developed by AstraZeneca. Although no thrombosis was demonstrated by imaging studies, she had very highly elevated d-dimer levels during hospitalization. Serology for antibodies against platelet factor 4 was positive on several days with very high optical density readings. We found that the antibody could induce spontaneous platelet aggregation without the presence of heparin. We decided to treat her with intravenous immunoglobulin, high-dose dexamethasone, and a prophylactic dose of apixaban. She improved rapidly and was discharged from the hospital 6 days after admission. Neither thrombocytopenia nor thrombosis was subsequently detected at the three-week follow-up.

**Conclusions:**

Despite the lower rate of thrombosis, VITT can occur in the Asian population. Early detection and prompt treatment of VITT can improve the patient’s clinical outcome. Thromboprophylaxis with nonheparin anticoagulants also prevents clot formation.

## Background

The Coronavirus Disease 2019 (COVID-19) pandemic has affected health and economic systems globally. Shortly after the first case series was reported in China in December 2019 [1], the number of new cases increased exponentially. COVID-19 is a serious infectious disease with a high mortality rate of up to 2% among infected patients [2]. Although the disease can be controlled by social distancing and wearing masks and face shields, immunization with a vaccine against severe acute respiratory syndrome coronavirus 2 (SARS-CoV-2) should be the method of choice to combat the COVID-19 pandemic. Four different types of vaccines are currently available [3], and vaccination was started in March 2021, with most adverse event reports indicating minor side effects. However, several groups of people receiving the ChAdOx1 nCoV-19 vaccine, an engineered nonreplicating viral vector vaccine using an adenovirus developed by AstraZeneca, developed a vaccine type-specific complication named vaccine-induced immune thrombotic thrombocytopenia (VITT). Patients usually develop thrombocytopenia and thrombosis [4–6] within 4–28 days after the first dose of vaccine [4–6]. Most of the cases were among young and previously healthy females [4–6]. Up to 38–80% of reported cases had severe thrombosis in the venous sinus system of the brain [4–6]. The mortality rate of VITT was high and reached 18% (71 of 390) in the United Kingdom, the country with the highest number of reported cases in the world [7]. Although the association between adenovirus viral vector vaccines and thrombocytopenia along with thrombosis is uncertain, it is believed that certain components of the vaccine could induce platelet aggregation and cause thrombocytopenia, eventually leading to thrombosis [6]. Moreover, it has been assumed that the mechanisms of VITT and autoimmune/spontaneous heparin-induced thrombocytopenia/thrombosis (HIT/T) are similar [6]. Although VITT has been mostly reported in the Caucasian population, there have been no reports in an Asian population receiving this type of vaccine. In Thailand, the vaccination program for COVID-19 was launched in May 2021, and mass vaccination with the ChAdOx1 nCoV-19 vaccine developed by AstraZeneca has been gradually rolled out to the Thai population since June 2021. Shortly after the start of this roll out, the first patient with VITT in Thailand presented to our institution.

## Case presentation

A 26-year-old female presented with severe headache for 5 days. She had been previously healthy without comorbidities 8 days earlier when she had been scheduled for the first dose of ChAdOx1 nCoV-19 vaccine (AstraZeneca) vaccination. She had no adverse events following immunization until 3 days later, when she developed a severe headache. She did not report any fever, myalgia, blurred vision, nausea, or vomiting. Her headache was not improved by acetaminophen and mefenamic acid. One day prior to admission, she noticed multiple discrete reddish spots on both legs without bleeding gums or epistaxis. On examination, mildly pale conjunctiva and petechiae on both legs were noted, while other examinations were unremarkable. The initial complete blood count showed a hemoglobin of 9.7 g/dL, mean corpuscular volume of 71.5 fL, white blood cell count of 3.66 × 10^9^/L (neutrophil 75%, lymphocyte 18%, monocyte 4.7%, eosinophil 2% basophil 0.3%), and platelet count of 22 × 10^9^/L. The prothrombin time was 11.9 s (normal range, 9.8–12.9 s), the activated partial thromboplastin time was 25.8 s (normal range, 21.8–30.2 s), and the fibrinogen level was 173.8 mg/dL. D-dimer was 9452 ng/mL (normal d-dimer, < 500 ng/mL). NS-1 antigen, dengue IgM, and IgG serologic testing for the diagnosis of dengue hemorrhagic fever were negative. SARS-CoV2 RNA test was negative. Lupus anticoagulants, anticardiolipin IgM, IgG, and anti-β_2_ glycoprotein I IgM and IgG were negative. Magnetic resonance imaging, angiography, and venography of the brain were normal without evidence of thrombosis. Computed tomography angiography of the pulmonary arteries showed no pulmonary embolism, and computed tomography of the abdomen was unremarkable.

**Fig. 1:**
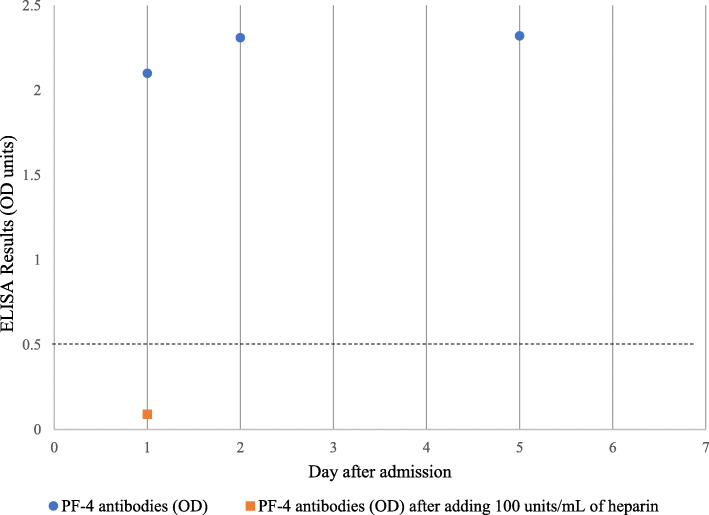
Summary of platelet factor 4 antibody results of the patient

**Fig. 2:**
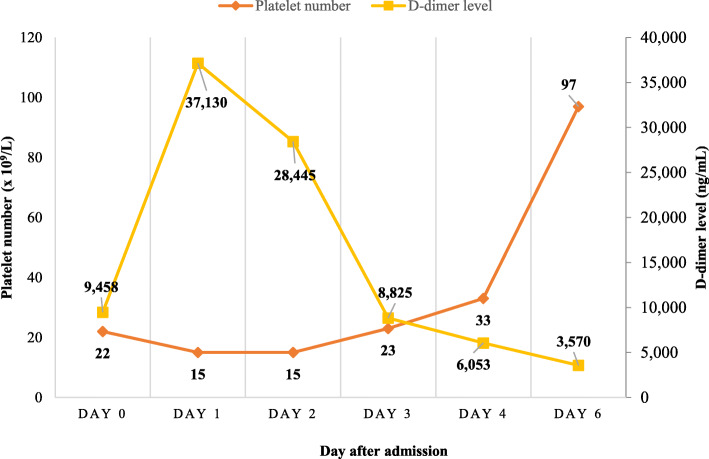
Timeline of platelet count and d-dimer level of patient

Due to the high degree of suspicion of VITT, we performed a test for antibody against platelet factor 4 (PF4) by enzyme-linked immunosorbent assay-based assay (Zymutest HIA IgG, HYPHEN BioMed, Neuville-sur-Oise, France), and the result was positive with an optical density (OD) of 2.10 (normal OD, < 0.4) (Fig. 1). After we added heparin at a concentration of 100 units/mL, the OD was lower at 0.09 (Fig. 1). We then demonstrated a functional test of antibodies by a heparin-induced platelet aggregation (HIPA) test. Platelet-rich plasma from a healthy volunteer with blood Group O was incubated with the patient’s serum for 1 h. Low doses (0.1 and 0.5 unit/mL) and a high dose (100 units/mL) of heparin and normal saline were added to the mixture. Platelet aggregation was assessed by light transmission (AggRAM Analyzer®, Helena Laboratories, Beaumont, Texas). After adding 0.1 and 0.5 units of heparin for final concentrations, 31.8 and 46% platelet aggregation was detected, respectively. After adding high-dose heparin (100 units of heparin for final concentration), platelet aggregation was 15.9%. Interestingly, spontaneous aggregation (30% aggregation) was found after saline was added instead of heparin (Table 1). After discussion with the patient, we promptly started treatment with 2 days (Day 1- Day 2 of hospital admission) of 1 g/kg intravenous immunoglobulin (IVIG) infusion and dexamethasone 40 mg per day for 4 days (Day 1- Day 4 of hospital admission). Apixaban was given to the patient beginning on Day 1 of hospital admission, and we planned to extend it for a total of 3 months. Despite a low platelet count, we were strongly against platelet transfusion, which may have worsened any thrombosis in the patient. We opted to monitor thrombus formation at the d-dimer level because we did not find evidence of thrombosis by imaging studies, and the d-dimer level gradually decreased during the hospital course after treatment (Fig. 2). After 6 days of IVIG and dexamethasone treatment, the platelet count slowly increased to 97 × 10^9^/L (Fig. 2). The patient’s symptoms improved, and she was discharged from the hospital on Day 6. At the three-week follow-up, her platelet count was 134 × 10^9^/L (Fig. 2), and no clinical thrombosis was detected while she was taking 5 mg per day of apixaban.

**Table 1:** Heparin-induce platelet aggregation results of the patient

Heparin for final concentration, units/mL	Platelet aggregation, %		
	D0	D1	D4
**0.1**	31.8	18.7	16.8
**0.5**	46.0	20.4	15.3
**100**	15.9	19.2	18.7
**Saline**	30.0	16.0	6.9

Abbreviations: *D#* Day after admission

Because our patient had a serious adverse event and it was a reaction to a COVID-19 vaccine, we reported our case to the National Immunization Center of the Thai Ministry of Public Health. Currently, there are three reporting systems in Thailand: the AEFI (Adverse Events Following Immunization) surveillance system, which is a passive surveillance system reported to by medical personnel; the Active Surveillance System for COVID-19 Vaccine, which is an active surveillance system reported to by vaccine receivers; and the Adverse Event of Special Interest (AESI), which is a passive surveillance system reported to by specialists in university hospitals. Reported adverse events from these three systems are sent to the National Immunization Center of the Thai Ministry of Public Health.

## Discussion

Since the first three independent reports of case series were published in April 2021, the number of patients diagnosed with VITT associated with the ChAdOx1 nCoV-19 vaccine developed by AstraZeneca has grown and continues to grow [4–6]. The Ad26.COV2.S vaccine, developed by Johnson and Johnson with similar adenovirus viral vector technology, was also reported to induce thrombocytopenia and thrombosis in the United States [8]. The ChAdOx1 nCoV-19 vaccine is a WHO-approved vaccine against SAR-CoV-2 and is widely used in Thailand. After the mass vaccination campaign was launched in May 2021, approximately two million doses of the ChAdOx1 nCoV-19 vaccine were administered. Thus, the incidence rate of VITT in the Thai population is approximately one in two million vaccinations, which is much lower than that reported in Caucasian populations [9]. There are several possible explanations for the lower incidence of VITT in the Thai population. First, Asian ethnicity is widely recognized to be associated with fewer thrombotic events than Caucasian ethnicity [10]. This could be due to a lower rate of inherited thrombophilia, such as prothrombin G20210A mutation or Factor V Leiden in Asian ethnicity [11], and Asian people requiring lower doses of warfarin treatment might imply an antithrombotic tendency [12]. Second, many reported cases of VITT had concomitant risks for thrombosis, such as antiphospholipid syndrome or oral contraceptive pill usage, which was not found in our patient.

Most patients with VITT present with an unusual site of thrombosis, such as the cerebral venous sinus or the splanchnic vein, with concomitant severe thrombocytopenia [4–6, 8]. The onset of VITT is usually 4–28 days after vaccination [4–6, 8]. Although the pathogenesis of VITT is uncertain, there have been many laboratory findings supporting the hypothesis that the mechanism of thrombocytopenia and thrombosis is similar to that of autoimmune HIT/T in that the anti-PF4 antibody may be induced by polyanions, including lipid A, in bacterial surface nucleic acids instead of heparin [13]. For VITT, some components of the vaccine, for example, the adenovirus DNA, spike protein, and/or neoantigen induced by the vaccine, have been proposed to be key components that could induce PF4 release and anti-PF4 antibody production [14].

To the best of our knowledge, our patient is the first reported case of VITT in an Asian population, and she had severe thrombocytopenia 8 days after ChAdOx1 nCoV-19 vaccine administration. Pathologic anti-PF4 antibody was demonstrated by high OD and subsequently confirmed by functional HIPA test. Although most VITT cases developed thrombosis, as demonstrated by either clinical features or imaging studies, our patient did not have thrombosis after extensive investigations for occult clots. This might have been due to the high degree of suspicion and the early detection of VITT because some patients with VITT who come to the hospital early may not have clinical thrombosis despite having a high level of d-dimer detected [15]. Although a rare side effect of immunoglobulin is thrombosis, IVIG is still an essential treatment to slow the progression of this disease [16]. Immediate treatment with IVIG can decelerate the progression of the immune reaction and accelerate the recovery of the platelet count [17]. Many guidelines also recommend starting treatment with IVIG in every case that is suspected of VITT without delay [18–20]. With lessons learned from our patient, we strongly support the use of anticoagulants other than heparin to prevent clot formation. Furthermore, the d-dimer level can be used to monitor thrombosis progression. Prophylactic anticoagulant for a duration of 3 months is recommended in patients with VITT [20].

## Conclusions

Our case report highlights that VITT can occur in Asian populations. Prompt treatment with IVIG, dexamethasone, and prophylactic anticoagulants can improve the patient’s outcome. More research data, especially about the risk factors and pathogenesis of the syndrome, are needed to encourage patients to be vaccinated confidently.

## Data Availability

Not applicable.

## Abbreviations

COVID-19: Coronavirus Disease 2019
HIPA: Heparin-induced platelet aggregation
HIT/T: Heparin-induced thrombocytopenia/thrombosis
IVIG: Intravenous immunoglobulin
OD: Optical density
PF4: Platelet Factor 4
VITT: Vaccine-induced immune thrombotic thrombocytopenia
SARS-CoV-2: Severe Acute Respiratory Syndrome Coronavirus 2
